# EDIN Scale Implemented by Gestational Age for Pain Assessment in Preterms: A Prospective Study

**DOI:** 10.1155/2017/9253710

**Published:** 2017-02-08

**Authors:** G. Raffaeli, G. Cristofori, B. Befani, A. De Carli, G. Cavallaro, M. Fumagalli, L. Plevani, F. Mosca

**Affiliations:** NICU, Fondazione IRCCS Cà Granda Ospedale Maggiore Policlinico, Università degli Studi di Milano, Milan, Italy

## Abstract

*Background.* Chronic neonatal pain can lead to long-term adverse effects on the immature brain. EDIN scale for prolonged pain might not be fully suitable for premature infants. We aimed to test a modified EDIN scale, adding postmenstrual age (PMA) as a sixth item (EDIN6).* Methods.* In a two-phase prospective study, pain was assessed in all neonates admitted in our NICU. In *T*1 EDIN was applied; in *T*2 EDIN6 with additional scores of 2, 1, and 0, respectively, for 25–32, 33–37, and >37 weeks PCA was tested. Scores > 6 suggested pain. The nursing staff was given a questionnaire to evaluate EDIN and EDIN6.* Results.* A total of 15960 pain assessments were recorded (8693 in *T*1; 7267 in *T*2). With EDIN6, cumulative detection of pain almost tripled (117/7267 versus 52/8693, *p* = 0.001). Main differences were found among less mature categories (50/1472 versus 17/1734, *p* = 0.001 in PCA 25–32; 26/2606 versus 10/4335, *p* = 0.001 in PMA 33–37; 41/3189 versus 25/2624, *p* = 0.26 in PMA > 37). Adequacy of pain assessment in lower PMA was judged “medium-high” in 13,4% of nurses in *T*1 and 71,4% in *T*2.* Conclusions.* EDIN6 may allow improved evaluation of pain in preterm infants.

## 1. Introduction

In the 1980s Anand and Hickey have described for the first time the potential mechanisms, by which neonates experience pain [1, 2]. Since then, progress has been made in understanding and treating neonatal pain [2, 3]. There is a growing concern for potential adverse long-term effects of recurrent pain on the developing brain, altering pain responses later in life [4]. Neuroradiological studies have proven the relation between early pain exposure in preterm babies and impaired brain development, in terms of size [5] and architecture [6] of the brain. Although the literature has been prolific on the topic, proposing many validated tools for neonatal pain assessment, most of them focus on acute and procedural pain [7]. A gold-standard measure for chronic pain is still lacking [8]. Currently, alternative available scales for chronic pain are, among others, EDIN [9], N-PASS [10], and COMFORTneo [11].

EDIN scale (Échelle de Douleur et d'Inconfort du Nouveau-né) is a one-dimensional behavioral scale, based on the assessment of 5 items: (1) facial expression, (2) body movements, (3) quality of sleep, (4) quality of contact with nurses, and (5) consolability; each of them is scored from 0 to 3; EDIN scores greater than 6 are considered expression of pain. This scale is easily applicable, as it does not require specific training [9]. Behavioral indicators may be affected by many factors, such as gestational age (GA), postmenstrual age (PMA), illness severity, continuous sedation, and cumulative experience of previous invasive procedures, leading to “sensitization” and “hyperalgesia” phenomena [12–14]. Moreover, when pain persists, physiologic indicators (such as heart rate, blood pressure, oxygen saturation, and breathing pattern) could stay unchanged, and reactions to prolonged pain could be more subtle and challenging to recognize, especially in the preterm baby [15]. All these factors may contribute to underestimation and undertreatment of pain [8, 15]. Among these aspects, age significantly seems to affect pain expression, altering basic behavioral state (asleep/awake) of neonates and their response to pain [16]. In 2009 Ancora and coworkers retrospectively analyzed the impact of GA and PMA on the expression of prolonged pain assessed by EDIN scale [16]. They found a relationship between GA and EDIN scores, reporting higher EDIN scores with increasing GA. More mature babies were thought to be more able to express pain if compared to their less mature counterparts. The authors concluded that the validity of the EDIN scale could be improved by taking into account GA and by attributing higher basal scores to more premature neonates.

*Aim.* In the present study, we tested a modified EDIN scale, named “EDIN6”. PMA was added as a sixth, nonbehavioral item. Our aim was to evaluate prospectively how the use of such an implemented scale could improve the management of chronic pain in critically ill neonates. The second research question addressed neonatal nurses' perceptions of reliability and clinical utility of EDIN6 versus EDIN scale in neonatal pain assessment.

## 2. Materials and Methods

This prospective observational study was performed between 24th November 2013 and 23rd March 2014 at our institution, Fondazione IRCCS Ospedale Maggiore Cà Granda in Milan. All neonates admitted to the 23-bed Neonatal Intensive Care Unit (NICU) and 33-bed Medium Care in that period were eligible. The patient population included term and preterm neonates of all GA, with both medical and surgical diseases and variable lengths of stay (LOS). No exclusion criteria were considered. The methodology followed the international guidelines for the observational studies is called STROBE [17].

By the Italian guidelines and recommendations for prevention and treatment of neonatal pain [18], in our NICU prolonged pain is routinely assessed by nursing staff, at least once during the observation period of a shift, using EDIN scale. The frequency of assessment may be increased, depending on ongoing analgesic therapy or painful events. The score is reported in each patient's electronic medical chart (Neocare®), to tailor intensive care. A total score > 6 is indicative of pain. Throughout the study period, analgosedation was provided according to hospital-based protocols. Nonpharmacological interventions consisted of sensorial saturation, sucking, individualized developmental care, environmental care, wrapping, and parental presence [18]. The pharmacological approach included paracetamol (mild to moderate pain) and opioids such as morphine and fentanyl (for moderate to severe pain).

We were relieved from the need for approval from the Institutional Review Board because of the noninvasive and purely observational character of the study. However, parents were informed of the ongoing study, and a written parental consent was collected.

### 2.1. Procedure and Timing of the Study

In November 2013, in a definite time (*t*0), a structured researcher-developed questionnaire (see Appendix), designed to evaluate the clinical utility of EDIN scale, was administered to participating nurses.

From November 2013, during a period of 60 days (*t*1), EDIN scores were obtained for each patient and collected in the electronic medical charts (Neocare). For each EDIN score > 6, data concerning frequency and modality of additional analgesic treatment were recorded.

From January 2014 to March 2014, for 60 days (*t*2), EDIN6 scale was applied, following the same procedure as in *t*1.

At the end of this second period, in March 2014, in a definite time (*t*3), a similar questionnaire (see Appendix) was administered to the nursing staff, evaluating EDIN6's performance.

### 2.2. Scales

EDIN was applied in *t*1, while EDIN6 was applied in *t*2. The latter was modified from the EDIN scale, by introducing PMA as an additional item, according to the analysis conducted by Ancora et al. [16]. This change provided two additional points to the PMA < 33 weeks, one additional point for PMA between 33 and 37 weeks, and no additional points for PMA > 37 weeks (Table 1).

Interrater reliability was calculated for 10% of the cases in both populations (*t*1 and *t*2).

### 2.3. Questionnaires

The nursing staff was administered a researcher-developed questionnaire, to assess caregivers' perceptions about EDIN (in *t*0) and EDIN6 (in *t*3) as pain assessment tools. Both versions (see Appendix) consist of eleven questions, divided into two main sections: the first one is identical for EDIN and EDIN6, while the second is scale specific. Questionnaires were developed according to criteria described by Boynton and Greenhalgh [19]. Staff nurses ranked each study tool using a Likert scale between 1 (not useful) and 5 (very useful). A convenient sample of participants included in the survey consisted of neonatal nurses, adequately trained to recognize neonatal pain through appropriate pain tools. Participants' demographic characteristics (educational background and NICU experience) were collected.

### 2.4. Patients' Data

Demographic and medical data were collected for every patient, by accessing electronic medical charts (Neocare). Data included gender, GA, birth weight, number of scores indicative of pain (score > 6), and modality of analgesic intervention in case of pain (score > 6).

### 2.5. Data Analysis

The two cohorts of neonates, respectively, evaluated in *t*1 and *t*2, were compared regarding patient characteristics at birth with Student's *t*-test. Answers to questionnaires related to EDIN scale and EDIN6, administered to nurses, respectively, in *t*0 and *t*3, were expressed as percentages of answers based on a 5-point Likert scale (very low, low, medium, high, and very high) and, at a later stage, they were compared using Fisher's exact test. Statistical significance was set at the 0.05 level. All statistical analyses were performed using Sigmastat Excel for Windows.

## 3. Results

### 3.1. Patient Population-EDIN Score

**Figure 1:**
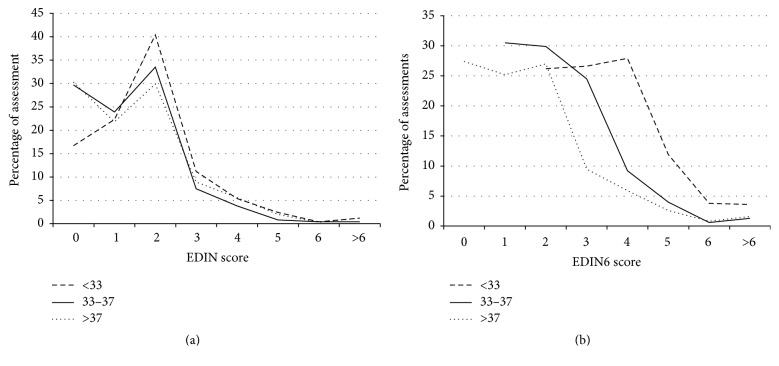
Percentage of pain assessment by (a) EDIN and (b) EDIN6 for PMA < 33 wks (dashed line); PMA 33–37 wks (continuous line); and PMA > 37 wks (dotted line). The majority of scores (99.4% in *t*1; 98,4% in *t*2) are indicative of “No-pain” state by all different PMA groups. EDIN: Échelle de Douleur et d'Inconfort du Nouveau-né; PMA: postmenstrual age.

During the study period, all neonates admitted to the NICU and Medium Care were included in the survey. Demographic and clinical data related to the two cohorts are shown in Table 2. In *t*1 EDIN scale was applied to 195 neonates, for a total number of 9193 assessments. 1844 (20% of the records) were performed in neonates with PMA < 33 wks (1st group); 4517 (49%) in neonates with PMA 33–37 wks (2nd group); and 2832 (31%) in neonates with PMA > 37 wks (3rd group). Edin scores > 6 detected pain only in 55 out of 9193 records (0,6%) and were almost equally distributed in the population: 17 assessments were related to the 1st group (0,9%), 10 assessments to the 2nd group (0,2%), and 25 assessments to the 3rd group (0,9%). As score > 6 suggests prolonged pain, analgesic interventions, both pharmacological and nonpharmacological, were recorded. An extra analgesic pharmacological strategy was adopted only in 22 cases (40%), 12 of which in the 1st group, 1 in the 2nd group, and 9 in the 3rd group. In *t*2 pain was assessed with EDIN6 in 138 neonates, for a total number of 7267 scores. 1478 of the records (20%) were performed in the 1st group; 2595 (36%) in the 2nd group; and 3200 (44%) in the 3rd group. As for the first cohort, distribution of scores remained asymmetrical, in every group studied. However, in the second cohort, EDIN6 basal scores were higher in the 1st and 2nd group, while they remained similar in the 3rd group. Score > 6 was recorded only in 117 assessments out of 7267 (1,6%): 50 reports in PMA < 33 wks (3,3%), 26 reports in PMA 33–37 wks (0,9%), and 41 reports in PMA > 37 wks (1,2%); see Table 3. As before, the association between score > 6 and analgesic intervention was recorded. Although EDIN6 scores > 6 almost doubled, if compared to EDIN ones, in group 1 (3,3% versus 0,9%, *p* = 0,001) and in group 2 (0,9% versus 0,2%, *p* = 0,001), the absolute number of observations indicative of pain remained low in both of the study periods (55–0,6% in *T*1, 117–1,5% in *T*2). Extra control-pain pharmacological measures were necessary only in 27 cases, corresponding to the 23% of prolonged pain conditions (10 interventions in the 1st group, 8 interventions in the 2nd group, and 9 interventions in the 3rd group).  Figure 1 shows a comparison of pain scores by EDIN and EDIN6 for different PMA categories.

Interrater reliability was very good within both periods (Cohen's Kappa was 0,82 and 0,86 in periods *T*1 and *T*2, respectively).

### 3.2. Nursing Staff Survey

**Figure 2:**
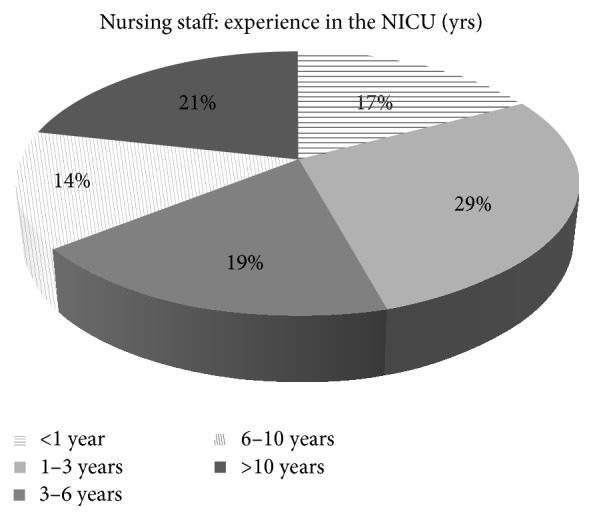
Variable clinical experience in the neonatal intensive care (years) of the nursing staff participating in the survey.

A total of 70 neonatal nurses participated in the survey. All were specifically trained for neonatal pain management, although with a variable clinical experience (see Figure 2). Results from scale evaluation through the five Likert-based questionnaires are summarized in Table 4. As shown, nurses highly appreciated EDIN6 introduction in clinical practice.

Part of the study results was presented at the Congress of Joint European Neonatal Societies, Budapest, 16th–20th 2015 (jENS 2015) [20].

## 4. Discussion

The neurophysiological basis of biological readiness to experience pain early in fetal development is well known [1]. However, response to pain may vary markedly across the age range of prematurity because of incomplete motor mechanisms required to communicate discomfort to caregivers [21, 22]. Many algometric scales have been introduced as a tool to recognize neonatal pain, both acute and chronic. Identifying prolonged pain is clinically relevant as it interferes with growth and altered pain response later in life [23–25]. Pain-related stress in newborns, especially in preterm babies, has been shown to impact negatively on brain development and to alter neuroendocrine stress response (hypothalamic-pituitary-adrenal axis), leading to cognitive and behavioral impairment [26–29]. Recently, human epigenetic research has focused on the association between early pain experience in preterm babies and behavioral impairment later in life [30]. Epigenetics seem to have a role, by altering the transcriptional functionality of the serotonin transporter gene* (SLC6A4 methylation)*, which was found to be predictive of infant temperament at 3 months of age [31]. Although research has focused on developmental nociception [32], networks and pathways involved in pain perception are not completely unveiled [8]. As a consequence, pain assessment methods are still rudimentary [8]. Among the available validated tools for acute/prolonged neonatal pain assessment, N-PASS and COMFORTneo are valuable options. The latter is a monodimensional behavioral scale to evaluate both pain and distress in the NICU. It was modified from the COMFORT scale, showing preliminary reliability [11]. Conversely, N-PASS is a multimodal scale and takes into account both physiological and behavioral items. It is actually the only scale to investigate vital signs in the context of prolonged pain. Additional points for prematurity are provided, in order to level out their pain response to full-term infants [10]. EDIN scale does not take into account GA [9], which is a relevant developmental determinant in pain perception, as it represents neonatal maturity [33, 34]. Ancora et al. provided evidence on the influence of GA and PMA on pain expression with an impact on EDIN score [16]. In the present prospective observational study, we investigated whether introducing GA as a sixth item to assess pain could effectively improve prolonged pain measurement in preterm infants. Our results from the first phase of the study (*t*1) show a complete overlapping distribution of scores for the three categories of GA. Moreover, 99.4% of the scores obtained continuously in 60 days of data collection (*t*1) are indicative of the absence of pain for all different GA groups (see Figure 1). These data are not comparable to those from the previous study by Ancora et al. [16], as we did not perform a multivariate analysis. From our results we can make two assumptions: firstly we could state that both the current “pain protocol” based on pain guidelines of the Italian Society of Neonatology [18] and analgosedation approach for each hospitalized neonate are appropriate to maintain a state of comfort in most cases. This may in part be explained by the increasing healthcare awareness and commitment to prevent and manage neonatal pain, as a result of a quality improvement training program. Secondly, we could speculate that EDIN scale, applied as proposed by Debillon et al. [9], might not be fully adequate to assess prolonged pain in preterm infants. Some of its specific behavioral items (quality of sleep or contact with nurses) are not straightforward but rather hard to identify in the immature neonates. Despite that, they represent pivotal elements for a comprehensive evaluation, especially in the hands of trained staff [9]. On the other hand, GA could be a missing key item in the EDIN assessment of neonatal pain. It is easy to add in an objective way, with no need for further time-consuming analysis.

In the second phase of the study the three categories of GA showed a different distribution of pain score; as expected, the more premature were the babies the higher were the basal scores. Nevertheless, even in the second period, the majority of scores were indicative of “No-pain” state by all different PMA groups. This fact probably happened because most babies had a very low basal score of EDIN and, even augmented by the points of the prematurity, they did not reach the level indicative of pain (>6). However, in *t*2 the percentages of preterm babies with a score indicative of pain were significantly higher than in *t*1; we conclude that the use of EDIN6 allowed us to point out the presence of preterm babies (fortunately few) who still needed an additional analgesic intervention. This explanation is strengthened by bedside nurses' judgment, although subjective itself, which strongly advocated for EDIN6 use.

In case of pain (score > 6), nonpharmacological measures were employed in most cases. The need for pharmacological additive intervention, always supported by medical advice, was only 40% in *t*1 and 23% in *t*2.

*Limitations.* The study population is very heterogeneous, as no exclusion criteria were considered. Surgical patients, especially those undergoing thoracic/abdominal surgery, are more exposed to pain than other patients. Moreover, different LOS can add further dishomogeneity, as well as the inclusion of the first day of life, which could lead itself to potential confounding stimuli, related to the delivery and early postnatal phase. Nevertheless, we believe that a significant number of term and preterm infants included in the study could be highly representative of NICU patients. Moreover, the possibility to survey a nursing staff, which was adequately trained for neonatal pain assessment, is another strength of the study, providing valuable insights into user's perspective.

As a proper management of neonatal pain is imperative to improve neonatal clinical practice, searching for a reliable measure of infant pain, especially of that born preterm, remains a challenge. Further studies are needed to identify a “gold standard” chronic pain assessment tool, suitable for the lowest GA groups.

## 5. Conclusions

We reported the results of the implementation in our NICU of the EDIN6 scale, a modified EDIN scale specifically designed to evaluate prolonged pain in different PMA categories of prematurity. Compared to EDIN scale, EDIN6 seems a valuable integrative tool in the management of preterm babies. Nursing staff perceived EDIN6 to be more suitable for pain assessment in the less mature infants.

## Figures and Tables

**Table 1:** EDIN6 scale (modified by Debillon 2001: Échelle Douleur Inconfort Nouveau-Né), integrated by gestational age as a sixth item.

Item	Description	Score
(1) Facial activity	Relaxed facial activity	0
	Transient grimaces with frowning, lip purse, and chin quiver	1
	Frequent grimaces, lasting grimaces	2
	Permanent grimaces resembling crying or blank face	3

(2) Body movements	Relaxed body movements	0
	Transient agitation, often quiet	1
	Frequent agitation but can be calmed down	2
	Permanent agitation with contraction of fingers and toes and hypertonia of limbs or infrequent, slow movements and prostration	3

(3) Quality of sleep	Falls asleep easily	0
	Falls asleep with difficulty	1
	Frequent, spontaneous arousals, independent of nursing, restless sleep	2
	Sleepless	3

(4) Quality of contact with nurses	Smiles, attentive to voice	0
	Transient apprehension during interactions with nurses	1
	Difficulty communicating with nurses. Cries in response to minor stimulation	2
	Refuses to communicate with nurses. No interpersonal rapport. Moans without stimulation	3

(5) Consolability	Quiet, total relaxation	0
	Calms down quickly in response to stroking or voice or with sucking	1
	Calms down with difficulty	2
	Disconsolate. Sucks desperately	3

(6) Postmenstrual age	Gestational age > 37 wks	0
	Gestational age 33–37 wks	1
	Gestational age < 33 wks	2

**Table 2:** Demographic data related to the group studied in *t*1 and *t*2.

	*T*1 (*n* = 195)	*T*2 (*n* = 138)	*p*
Gestational age, mean (ds)	35,1 (±4)	35,2 (±3,5)	0.91
Birth weight, mean (ds)	2340 g (±919 g)	2302 g (±860 g)	0.69
Gender (M/F)	107/88	71/67	0.58

M: male; F: female.

**Table 3:** Results expressed as score indicative of pain (>6)/total number of evaluation.

	EDIN (%)	EDIN6 (%)	*p*
Gestational age < 33 wks	17/1734 (0,9)	50/1472 (3,3)	**<0.001**
Gestational age 33–37 wks	10/4335 (0,2)	26/2606 (1)	**<0.001**
Gestational age > 37 wks	25/2624 (0,9)	41/3189 (1,3)	0.26
Total population	52/8693 (0,5)	117/7267 (1,6)	**<0.001**

Pain detection = Score > 6/Total evaluation (%).

**Table 4:** EDIN6 versus EDIN. Comparison of answers to the questionnaire (*t*3 versus *t*0). Percentages of *opinion “medium,” “high,” and “very high” *(*%*)* are shown*.

	EDIN (%)	EDIN6 (%)	*p*
Convenience	49/70 (70)	64/70 (91,4)	0.002
Adequacy to assess neonatal pain in NICU	12/70 (17,1)	55/70 (78,5)	<0.001
Influence on nurse's analgesic strategy	14/70 (20)	70/70 (100)	<0.001
Influence on physician's analgesic strategy	10/70 (14,2)	46/70 (65,7)	<0.001
Adequacy to assess pain in PCA < 37	9/70 (12,8)	50/70 (71,4)	<0.001
Adequacy to assess pain in sedated neonates	16/70 (22,8)	50/70 (71,4)	<0.001
Adequacy to assess pain in predischarge neonate	55/70 (78,5)	61/70 (87,1)	0.26
Correlation with subjective perception	23/70 (46)	56/70 (80)	<0.001

## Appendix

### Questionnaire Administered to Nursing Staff (*n* = 70) for Evaluation of EDIN (*t*0) and EDIN6 (*t*3) Scale

1. How long have you been working in a Neonatal Intensive Care Unit?
  - <1 year
  - 1–3 years
  - 3–6 years
  - 6–10 years
  - >10 years
2. In your opinion, how would you evaluate your training on neonatal pain
  - very high
  - high
  - medium
  - low
  - very low
3. What is your knowledge of the neonatal pain management procedure of the NICU you're working in?
  - very high
  - high
  - medium
  - low
  - very low
4. In your opinion, is the scale useful?
  - very high
  - high
  - medium
  - low
  - very low
5. In your opinion, is the scale adequate for pain assessment in the NICU?
  - very high
  - high
  - medium
  - low
  - very low
6. In your opinion, how much the scale score influence nurse's management of neonatal pain
  - very high
  - high
  - medium
  - low
  - very low
7. In your opinion, how much the scale score influences physician's management of neonatal pain
  - very high
  - high
  - medium
  - low
  - very low
8. According to you, is the scale adequate for pain assessment in the preterm?
  - very high
  - high
  - medium
  - low
  - very low
9. According to you, is the scale adequate for pain assessment in a sedated patient?
  - very high
  - high
  - medium
  - low
  - very low
10. According to you, is the scale adequate for pain assessment in the pre-discharge phase?
  - very high
  - high
  - medium
  - low
  - very low
11. According to you, does the scale correspond to your subjective perception of the neonatal pain?
  - very high
  - high
  - medium
  - low
  - very low

## Abbreviations

GA: Gestational age
EDIN: Échelle de Douleur et d'Inconfort du Nouveau-né
LOS: Length of stay
NICU: Neonatal Intensive Care Unit
N-PASS: Neonatal Pain, Agitation, Sedation Scale
PMA: Postmenstrual age.
